# Modern Tendency of Homœopathy

**Published:** 1871

**Authors:** 


*Modern Tendency of Homœopathy.*

My Dear Professor—Some one recently asked for light on homœopathy; your October Journal doubtless contained it, but as it has been swallowed up in the great fire, I hereby propose to triturate homœopathy a little and to essay a picture of that attenuated fraud as it stands at present.

Homœopathists who wish to succeed now, find it necessary to be graduates of regular colleges, and further, they find it necessary to practice regular medicine, at least to some extent; complying with these requirements, they use the name homœopathy as an *open sesame* to the purses of the credulous, and often accomplish fortune.

There are those who still believe religiously in the doctrines of Hahnemann; yes, who believe in the infallible efficacy of potencies diluted to an extent of which Hahnemann never dreamed; they form the bulk of the "profession;" their gushing faith fills homœopathic journals, their enthusiastic conversation revives our confidence in the existence still of innocence and simplicity, but they don't *succeed;* the world has gotten beyond that point of credulity, it still seeks the names of these false gods but not their faith.

I have sought for good in homœopathy, have heard lectures upon it, read Hahnemann and other authorities, and, lastly, have interviewed the best homœopathist I could find, telling him that I wanted to find out if there were anything good in it, and if there were, to adopt it. I asked for a case of disease and homœopathic treatment; he gave intermittent, said that rhus, cinchona, etc., would produce similar symptoms in health.

Q. We use cinchona; what dose do you use it in?

A. "One drop of tincture in teaspoonful of water every hour."

Q. Does it break the chill?

A. "Yes, in a few days."

Q. Not at once?

A. "Sometimes it does at once, but nearly always in a few days."

Q. Ague is rare and mild here, we never fail to stop it at once, not letting patient have another chill.

A. "Rarity and mildness of ague admitted."

Q. You say you stop chills in a few days; we stop them at once with quinia; here we seem to have an advantage over you. How does an infinitesimal dose of cinchona act?

A. "I don't know; how does a large dose act?"

(*Argumentum ad hominem.*)

Ans. continued: "They theorize on the subject, but I think it is wrong to do so; they say dilution frees force, that division and trituration develop dynamics."

Q. This seems to be sticking to the doctrines of Hahnemann. Are his maxims still regarded as orthodox in your school?

A. "Yes, they are still the law, and dilutions are now carried much higher than they were by Hahnemann."

Q. Do you believe in these dilutions?

A. "I can scarcely believe in them."

"It is contended by some that the original drug contains a spiritual agent, which is the truly curative power of the drug, and is developed or set free by the triturating and shaking processes; others contend that the development of the curative powers of a drug is a purely mechanical thing, and that the mechanical breaking up of the constituent particles of the drug constitutes that development. By the former these successive developments of the original substance are called *dynamizations* or *potentizations ;* by the latter, *attenuations."—Jahr & Gruner's Homœopathic Pharmacopœia.*

(The above interview was with one of the latter class—an intelligent gentleman and excellent surgeon.)

Interview with one of the former, the more numerous class:

Dynamizationist. "We use many drugs that you do; instance, rhubarb in dysentery."

Q. Why do you use it?

A. "It produces symptoms of dysentery in health."

Philosophic reason; *cœli cœlorum!* Suppose that a man is suffering extensive perturbations in the dynamics that vibrate around the sigmoid flexure of the colon, having congestion there, caused by the presence of a load of green apples, shall we attempt a neutralizing perturbation of those dynamics with the microscopic fraction of a grain of rhubarb? or shall we remove those apples, correct the changed structure of the inflamed part, and cause pain to vanish with the sulphureted hydrogen, like the baseless fabric of a vision leaving not a rack behind?

Our homœopathic friends believe that force obtains more freedom and becomes therefore more potent as it is attenuated; for instance, a grain of zinc is an aggregation of material atoms, around each of which, atoms of force exist; they believe that if this grain of zinc could be dissolved in 100 drops of water that there would be less force in the solution than if one drop were taken from it and mixed with another 100 drops of water, and that this second "potency" would not be so potent as the third, and so on; this belief is indicated in almost every case recorded by homœopathists. If it were true, a current of force sufficient to work the Atlantic cable ought to be increased by attenuating the zinc in its battery, but methinks electricians would be somewhat astonished if told that attenuating their zinc would develop more dynamics. The Atlantic cable has been worked by a battery composed of a percussion cap, but, sad to say, the current was not any stronger than when 30 cups of *Grove* were used, but on the contrary, was weak exactly in proportion to the attenuation of the zinc, and I affirm that if zinc 30 were used, the amount of force given off would not be appreciated by a dynamometer if its delicacy were multiplied by all the figures in mathematics. Here is a fair test; if homœopathists can develop dynamics by attenuation, let them develop it in the form of electricity, or heat, or light, or any other form that can be appreciated; and here let us ask them what form of force do their potencies develop in the body? Is it nerve force, or electricity, or what? Force is a unit; there are differences in degree (of motion) not in kind (of force); these degrees are convertible by changing structure;* please then, dear dynamizationists, convert the force which you say zinc $\underline{30}$ produces in the body, into magnetism or *something*, and *increase* it as you attenuate your zinc, say to o $\underline{60}$ or o $\underline{200}$; failing to do this, will you forever hold, etc.? or will you still talk of Jolly's experiment with water and saltpetre? (in which water decreased in volume on the addition of said salt.) Grauvogl, in his text book of homœopathy, puts it in this light: If it requires 940 atmospheres to compress water, a solution of saltpetre is equal in force to 940 atmospheres, if it does the same work. This is as good as saying, that if it requires the blow of a sledge hammer to kill a man, a grain of arsenic is as strong as a horse if *it* kills a patient. "Like produces like," but how about the *like circumstances*, how about the essential relation? Is there any relation between the manner in which saltpetre acts and the action of 940 atmospheres in a pump?

But our friends decry chemistry, it is too gross for them, the human laboratory is the perfect thing for demonstrating the dynamics of the infinitesimal; casting aside all calculations based upon chemistry or mathematics, they appeal to experiment alone. Grauvogl says: "When I began the study of homœopathy, the rock of the attenuation question on which so many of our adversaries' heads have suffered shipwreck, was to me an object never worthy of consideration; * * * *. The laws touching the proportion between matter and vehicle interested me the less because I had first of all to discover by experiment and observation the laws of relation between these homœopathic attenuations and my own organism, hence I proved on myself, etc. * * * *; these experiments and observations had driven from me forever

* Under the laws of correlation of force; instance, battery producing heat or electricity by varying structure of zinc, and producing light by another slight change.

all doubts about the efficacy of homœopathic attenuations, I gave myself no further trouble about the well known mathematical description of these attenuations which were sufficient to frighten so many away."

Here is the final appeal, viz.: to experience, or should we not say to imagination, morbid attention, and sleeplessness?

Having thus seen our homœopathic friends place themselves in outer darkness, hiding their little arrangements from the light of chemistry as well as physiology, it only remains for us to investigate their last resort, experience, as recorded in their journals. And here I had intended to transcribe 17 or 18 cases from infallible homœopathic journals, taking them in rotation just as presented, to show that their treatment bore about as much relation to recovery as cunderango to the siege of Paris; but those transcriptions have now evaporated to the home of the fire fiend, so the brethren must read for themselves; and O, dear brethren, are our own skirts clear here? and will not some coming homœopath elevate *my* Mansard and furnish *me* with a gutta percha aural apparatus on the strength of "heroic opiates," "sedative purges," and other idolatries worshiped by our own routinists?

I have not thought it necessary to *satirize* homœopathy; that has been done so thoroughly that I rather feared plagiarism therein. I might have mentioned that salt hath 687 symptoms, that I find 100 symptoms given as a few of those of the larynx, with 500 arrangements of "remedies" *indicated* therefor; might have quoted "CIMEX LECTULARIUS (bedbug). We crush the insect while alive, and macerate it in alcohol."—*Jahr & Gruner's Hom. Pharmacopœia;* but mute horror prevents further progress in this direction. Homœopathy hath a dictionary of symptoms, it is an immense thing, they refer to it in every case; no exercise for reason or judgment, only for memory; no account is made of structure as influencing disease, only "perturbation of dynamics."

I have thus endeavored to photograph the prominent points of modern homœopathy, touching the attenuation question, for which I have suggested a test that I think will be appreciated by students of force and its manifestations, referring to experience, the final resort of homœopathists, upon which their journals are living satires, and indicating the tendency of the present time, which is for intelligent homœopathists to discard everything homœopathic but the name, the only good thing I can find in it, and the only advantage they have over us. I apprehend that homœopathy thus shown is not to be cured by persecution, nor yet to be killed by hugging it to death as some have recommended, but that it will perish of *inanition* as soon as we can apply a cure for the chronic stupidity of our own dogmatic empirics who are of drugs druggy, which cure is pathology, and I do not know, my dear professor, how we are to hasten this consummation unless you write a book upon the principles of medicine.

When all our physicians under the enlightened teaching of this age shall discard the mysterious and supernatural, and shall practice upon certain knowledge of "what is the matter," ceasing to give drugs because they are said to be "good for it," but using the active agents of the Materia Medica to produce definite results upon organic structure, or as exceptions, when overwhelming evidence attests their efficacy; then, I think, the success of physiological medicine will leave no room in public favor for such speculative follies as homœopathy.

Respectfully, J.

CHICAGO, Oct. 20, 1871.

---

DEAR PROFESSOR—The zeal of your aged correspondent in behalf of homœopathy is quite amusing; and as he seems to desire information as to why the regular practitioners (of the old school of Hippocrates and Sydenham, of common sense as well as science), are opposed to homœopathy, I will try and inform him. In the *Medical and Surgical Reporter* for April 1st, 1871, our antiquated friend will find the legal definition of a quack, by the Supreme Court of New York, to be, "Whoever offers to practice homœopathy or allopathy as his patients may wish, is practically a *quack* in his profession."

We commend this to the consideration of our aged friend and to those numerous doctors who "have studied both schools and find good in both," as they delight in informing their patients—*Ex pede Herculem.*

We will not attempt to reply to his arguments seriatim, but will try and give a general review of the subject in a familiar and colloquial style.

Firstly, of the system of homœopathy what most staggers our judgment is the infinitesimal smallness of the doses. And as to these minute doses, for the physical efficacy of which they vouch so manfully, I have a few things to object. They say that it is a well ascertained fact that the ten-thousandth part of a grain of antimony will produce an appreciable effect, as that a scruple will; or as certain as any fact in the range of inductive science. I doubt it; I can and must judge, principally, from my own consciousness, though not from that alone. I take your prescribed globule, and cannot find that it produces the slightest effect upon me. I am willing to take any of your decillionths of grains from one to fifty. I have done so, and I do not find the effects assigned follow from these minute elements, and this experience is universal. They may say that the experience of others is different; that they find the minute doses palpably "potential;" that the effects of even a decillionth of some substances have been appreciable. No such averments can annul the *negative* instances I have mentioned; for your inference on the positive side, may easily be the fallacy—"*Non causa pro causa.*"

For example, the peristaltic action is often slightly increased by the mere imagining that medicine has been taken when it has not; many other processes are quickened by fancy; in many, again, all that is required is, instead of taking medicine, to use a little patience, and nature will perform her wonted task without the *globules*, and will doubtless perform it none the less *because* of the globules.

I have known a patient troubled with insomnia take his invaluable "minutissimum" of a soporific—his narcotic atom—and congratulate himself next morning that, after only two hours or so of restlessness he fell into a calm sleep—all owing, of course, to the viaticum of a globule!

I, on the other hand, equally troubled with sleeplessness, perform the same feat perpetually without any globule at all.

The simple solution is, that both parties are wearied out and at last go to sleep. Now, I can account for the effects in many such cases, without supposing your globule has had anything to do with them; but I cannot account for the *want* of effect in the negative instances; that is, where your globules to all consciousness produce none. They may reply that there are cases in which large doses fail of their effects. I grant it; there are no doubt cases in which the effect is intercepted by special causes; but we must go by general *induction*, and five grains of opium, or two scruples of rhei, will effectually convince nine hundred and ninety-nine out of every thousand that they have taken something. The difference in the two cases is, that those who venture to say they are conscious of the effects of your decillionths are, so far as I know, very rare exceptions; while of those who take the larger doses, the rare exceptions are those who are not affected; that is the general rule, and the exceptions change places. Again, when the larger doses fail of their general effect they leave potent signs to consciousness that something has been taken; whereas I can take one or ten of your decillionths of a grain every hour for four and twenty hours together without any conscious effects whatever; and other people have similar obstinate experience.

Another doubt I feel as to your infinitesimal doses is this. How can you be sure that you have administered them—that they have got into the patient's stomach at all? If they have not got there, I admit they will produce no more effect than they usually do when they *have* got there. But I know not how to be sure that they have reached their destination. They may, like the globule which was arrested in the hollow tooth of Hahnemann's patient, (his solitary fatal case!) be waylaid by a million obstacles, each too much for the poor little atom.

Moreover, I cannot comprehend, on Hahnemann's theory, how we can remain in health a day, since we must be taking all day long, through our lungs and in our food (especially in these days of adulteration), your minute doses of the most deleterious substances. If they say, according to the usual assumption (and it is nothing more,) that they will only affect the person in disease, and not in health, then when he is out of health, positively ill, and under treatment, these potent though inappreciable agents must come into play, and I think must confound your therapeutics. If they say they all happily neutralize one another, I suppose your little globule will be but another element among them, and must, I think, get neutralized too: certainly you know as little what becomes of *it*, as of them.

At all events it is clear, that if such a chance medley of potent "infinitesimals" *can* thus happily neutralize one another, anything like a calculable administration of your solitary infinitesimal is out of the question.

One need not be surprised at the homœopathist, the contents of whose "medicine chest" his children got hold of, played with and jumbled together (all unknown to him), who went on practicing with the same success as before.

The oddest part of Hahnemann's absurd theory of "dynamization" is, that infinitesimal doses are not only potent, but potent in the ratio of their minuteness. According to this, the "second, third, fourth, etc., orders of infinitesimals" (as mathematicians would say), are progressively powerful; in proportion, it seems, as an atom becomes more attenuated—nearer to nothing, it becomes so much more efficacious! Just as it vanishes, I presume it must be—omnipotent!

Nothing can exceed this doctrine except it is Hegel's philosophical paradox: Nothing—Being. If their theory be true, I marvel at the usual language of the homœopathists, who speak of the higher dilutions in the order of feebleness, not of potency, and tell a patient not to venture in such a case on anything stronger than number thirty.

To be consistent, they ought rather to enlarge than to diminish their doses, where they wish to diminish the effect. Nay—surely a scruple of strychniæ ought to produce less effect than a grain—and a grain than a trecillionth of it.

But there is one statement in our venerable friend's communication which I cannot let pass without special comment. He says, "homœopathy has taught us two things, that sick people will recover with small amounts of medicine or none at all." Or, in other words, the public are indebted to the theory of minute doses for a modification in the practice of the "old school;" that it has abridged that wholesale exhibition of drugs which used to be the fashion, and which turned many a patient's stomach into a drug shop.

I am really pleased to believe that the rivalry between the medical factions has been attended with some such effects. At the same time do not flatter yourself that the revolution is greater than it is. Too much physic used to be given, that is certain, but do not suppose all was physic that was taken.

Rely on it—as many a medical man's confession, if ingenuous, would show us—that it was not left to the homœopathists to discover the virtues of a placebo, or in other words to find out the art of doing nothing, under the pretence of doing something, just to amuse a patient; *vixerunt fortes ante Agamemona;* millions of innocent draughts of infusions of roses—and drams of syrup, quite as harmless as your globules, used to travel down the throats of patients simply because they *would* have something, and—"*Populus vult decipi et decipietur.*"

The only difference between the two classes of practitioners often is, that the one charges in the direct proportion of the innocent bulky nothing, and your friends charge in the inverse proportion of the innocent infinitesimal nothing. It was, I grant, rather an absurd practice, but it was used only as a *dernier ressort*, for many patients would not be cured unless they swallowed all this nothing, and what is more important to the doctor, would not pay unless they had as they thought "value received" in the shape of the material drugs, instead of reckoning their true debt to his visits and his skill. But be assured, my dear "Senex," that the essence of this branch of the art—of doing nothing under imposing forms—was understood long before homœopathy was born, and will be understood as long as the credulity of patients shall demand that something shall be done, when the physician knows that nothing need be.

One of their negative arguments is, "If the globules do no good, they cannot, on *our* theory, do harm." I fear there are many cases, and I have seen some, where your globules have done much harm by preventing anything good being done;—where symptoms that required prompt treatment, were dawdled with until the disease got strength and it was too late to do anything. I must also express my conviction that your doctors have an incomparable knack at making hypochondriacs; and I think very naturally. How should it be otherwise? Your system teaches a patient to believe that his life is ever at the mercy of infinitesimal elements and infinitesimal changes. Can he be other than hypersensitive about matters which never trouble other people's sleep? Certainly, as far as I have observed, there are no people in the world who require the doctor, or take medicine so often as the devotees of homœopathy; hardly a day passes without the medicine chest. Well for them that it contains nothing!

Similarly, nobody is so sensitive about all kinds of innocent changes of air and diet. For my own part, it would be a torment to live on the terms of some of the votaries of your infinitesimal doses, whom I have known.

However, I admit that such individuals are to be met with among the patients of the regular practitioners, though I think your system is especially adapted to befool a nervous temperament and stimulate a morbid fancy. I handsomely concede that there are classes of patients to whom your practice may be beneficial.

Firstly, I think it is of admirable use for those patients—and there are many—*who have nothing the matter with them ;* for as they will take physic and require none, it is better they should take nothing though they think it something—at the same time it must be said, bread pills might on the other system do the work of nothing just as well. Secondly, for those who suffer from anomalous conditions of the nervous system, amenable in a measure to the fancy (as they often seem to arise from it), but whose symptoms baffle all rational treatment. It is often important that these patients be amused with the appearance of something being done—though here again the more bulky vehicles of *nothing* may do as well, for aught I can see, as the infinitesimals. Thirdly, for those who have, indeed, something the matter with them, but whose symptoms are so obscure that a wise physician is afraid to do anything lest he do mischief; while yet (as usual) the patient insists that something shall be done. Now here the globulets (if I may venture on the double diminutive) are admirable, I admit; though, again, the more corpulent pill of *micæ panis* may be just as efficacious.

In short, as long as patients are so credulous in their reliance upon medicine as to insist that when the doctor knows that nothing need be done, or can be done, he shall yet do something, I see no help but in the exhibition of a placebo. If it be gravely argued that it is unworthy of a physician to administer a system of delusion, and that he had better leave his patient uncured than cheat him into health, it is a pleasant question of casuistry, which the doctor may, if he chooses, discuss in a clinical lecture and see what his patient says to it.

It is in vain, my learned doctor, that you reiterate that you have "seen the good effects" of your darling globules—"that you have seen your nieces, nephews"—*ad infinitum*, recovered under their use.

I have no difficulty in believing any facts merely on account of their mystery, and if on a fair induction more patients were discovered to be cured by your system than any other, I should believe in it, were it (if that be possible) ten times as mysterious. But a single case or two, or any man's private experience, does not by any means entitle him to speak *ex cathedra* on the subject, and is not worth a fig in the controversy either way; for the simple reason that every system of medicine might be proved equally efficacious on the same ground.

The Wit and Poet of our profession, Professor O. W. Holmes, in a lecture some time since, made use of the following language, which is quite apropos: "What is the honest truth about the medical art? That by far the largest number of diseases which physicians are called to treat will get well at any rate, even in spite of reasonably bad treatment. That of the other fraction a certain number will inevitably die, whatever is done. That there remains a small margin of cases where the life of the patient depends on the skill of the physician. That drugs now and then save life; that they often shorten disease and remove symptoms; but that they are second in importance to food, air, temperature, and other hygienic influences."

A very common fallacy is, that of "*non causa pro causa*," and especially in medicine, where a plurality of causes or *apparent* causes may perpetually mislead.

To the generality of men, it is enough if a certain antecedent has preceded a certain consequent, to satisfy them that there is the relation of cause and effect. Hence numberless fantastic remedies which different ages and nations have prescribed as useful merely because their employment has happened to be nearly coincident with the cure, though they have no more caused it than the cock's crowing causes the sun to rise.

The credulous association of a mere antecedent of the cure with the cause of it, (which is almost universal with patients,) is, it must be allowed, too much encouraged, by doctors of all kinds. Nothing is more common in reports of cases than to find an improvement attributed undoubtingly to the administration of such a remedy, when the difficulty is really to establish the connection.

If the patient gets worse after the medicine, we never find this sequence insisted on; though, for anything we know, it might be just as reasonably. "Ah!" says a patient, "it was a good thing I called in the doctor; he cured me." If he is cured without any doctor at all, he thinks nothing of it.

When the patient recovers, the doctor gets rid of the disease in spite of nature; when the patient dies, nature gets rid of the patient in spite of the doctor! How do we know how often the statement ought to be reversed; how often nature saved the patient in spite of the doctor, and how often the doctor killed the patient in spite of nature.

You may infer that I am sceptical as to the use of medicine altogether; you will infer falsely then. I do believe that the great majority of ordinary attacks of disease would be cured without the aid of the physician, and that the great agent of cure is the *vis medicatrix*, with which our Creator has fenced the human organism, and by which it stoutly resists every incursion of disease.

But I believe there is a noble sphere for the physician, too; though I frankly confess that from the extreme difficulty of a really comprehensive induction—of establishing the true connection of antecedent and consequent, and from the infinitely variable, evanescent phenomena the science of the practice of medicine has to deal with—it will yet be many years before it will attain the certainty of an exact science.

Nevertheless, the wise physician has plenty to do—especially if he will not promise or attempt too much; if he will but be content to be the cautious *naturæ minister*, and stand by with the hope of aiding those processes within us, so many of which transcend all his art, and which, if he be rash, he may more easily hinder than help; or if, in a word, he takes that view of his position to which "old experience does attain," and which, in the language of Dr. Forbes, will lead him to acquiesce "in a mild tentative or *expectant* mode of practice," certain to appear wise "in old age, whatever may have been the vigorous or heroic doings of youth."

Surely we must allow that even if the physician only alleviates pain and abridges processes which might otherwise be tedious, he is well worth all his fees. Nor less if he takes charge of us in *health*, and, studying its general physiological conditions, endeavors to keep us well.

In truth, paradoxical as it may seem, it is in health that we ought chiefly to look to the physician and to avail ourselves of his skill. We should listen to what he says about how we are to keep out of his hands, about regimen, diet, hours, occupation, etc.; and the next best thing is, to consult him, not when we *are*, but when we are *going to be*, ill; when we are getting "out of health," as the phrase is.

As to the universal principle of homœopathy, I must say that the assertion of one "universal principle" on which all diseases are to be cured, (like "similia similibus curantur") has a mighty occult quackish sound, and looks more worthy Paracelsus than Bacon.

Neither is it quite fair for Hahnnemann to charge all other practitioners with uniformly proceeding on some one opposite principle, as "allopathy or antipathy," for neither homœopathy or allopathy was ever heard of till he chose to invent the terms, and, taking one himself, gave the other to all the rest of the medical world.

People may call themselves what they please, but if they apply a term to their neighbors, they should see that it is one that belongs to them.

The medical profession, as represented by the American Medical Association, for instance, or by the teachers in the leading universities and medical colleges of the country, are not "allopathists" at all; but if they must have a Greek name of this pattern, they are *pantopathists;* that is, they profess only this simple doctrine, *to employ any agency which experience shows to be useful in the treatment of disease.* Anything that can make a decent show for itself is sure of a trial at their hands.

Remember this, then, that the medical profession, fairly enough represented by the bodies I have mentioned, have no theory or doctrine which prevents them from using *anything that will do you good.*

If they do not adopt this or that alleged remedy it is because they do not think a case is made out in its favor. They consider the witnesses incompetent or dishonest, it may be, or the evidence wholly unsatisfactory on its own showing. Our friend seems anxious to borrow some remedies from the disciples of Hahnnemann. Does he remember how rapidly any real discovery is appropriated and comes into universal use? Take anæsthetics (mercifully granted to a world grown sensitive in proportion to its culture); take the use of the bromide of potassium; and see how easily they obtained acceptance.

If he is disposed to think any of the fancy systems has brought forward any new remedy of value, which the medical profession has been slow to accept, I ask him to name it. Let him name one—the best his system claims—a single new, efficient, trustworthy remedy, which the medical profession can test by the *experimentum crucis*, as they are ready to test before any scientific tribunal, opium, quinine, ether, bromide of potassium. There is no such remedy on which any of the fancy practitioners dare stake his reputation. If there were, it would long ago have been accepted, though it had been flowers of brimstone from the borders of Styx or Cocytus.

Pardon me, if, in conclusion, I just hint that the writing of defences of homœopathy, by a practitioner who claims to be a "regular," is, to say the least of it, rather quackish, and altogether uncalled for.

If our friend "Senex" was as zealous in behalf of legitimate medicine as he is for homœopathy, he would soon find himself *au courant* with the vanguard in the onward march of practical medicine, and far beyond the pale of such an *ignis fatuus* as homœopathy. For in his zeal he has become quite a homœopathic knight-errant or evangelist. But if I were a homœopathic doctor I should say of all such amateurs, "*Non tali auxilio.*"

Respectfully yours,

WILLIAM E. BRANDT, M.D.

HANOVER, INDIANA.

